# Development of A Surgical Treatment Algorithm for Breast Reconstruction in Poland Syndrome Patients Considering Severity, Sex, and BMI

**DOI:** 10.3390/jcm10194515

**Published:** 2021-09-29

**Authors:** Maximilian Mahrhofer, Thomas Schoeller, Maria Casari, Kathrin Bachleitner, Laurenz Weitgasser

**Affiliations:** 1 Department of Plastic and Reconstructive and Aesthetic Surgery, Marienhospital Stuttgart, Teaching Hospital of the Eberhard Karls University, 70199 Tuebingen, Germany; thomas.schoeller@vinzenz.de (T.S.); maria.casari@stud.pmu.ac.at (M.C.); kathrin.bachleitner@vinzenz.de (K.B.); laurenz.weitgasser@vinzenz.de (L.W.); 2 Paracelsus Medical University, 5020 Salzburg, Austria

**Keywords:** Poland syndrome, breast reconstruction, transverse myocutaneous gracilis, DIEP, TMG, TUG, free flap, microsurgery, lipofilling, breast implants

## Abstract

Introduction: Poland syndrome is a rare, challenging combination of chest wall and breast deformities for reconstructive surgeons and selecting the treatment can prove difficult. This study aims to help surgeons in choosing the best viable option for treatment by sharing our institutional experience and proposing a guiding algorithm. Methods: A retrospective analysis of all patients with Poland syndrome undergoing treatment for breast and chest wall deformities at a single institution between December 2011 and May 2020 was performed. Medical charts were reviewed to allow for a description of patient demographics, treatment modalities and complications. A treatment algorithm to aid in selecting the adequate reconstructive option based on our institutional experience was formulated. Results: A total of 22 patients (six male, 16 female) were identified who received treatment for Poland Syndrome related deformities. Nine received microsurgical free flap reconstruction (three Deep Inferior Epigastric Perforator flaps, six Transverse Myocutaneous Gracilis flaps), two received reconstruction with a local flap (two Latissimus dorsi flaps), nine received implant based reconstruction, and two were treated with autologous free fat transfer only (17 in combination with other surgical methods). Conclusion: Free flap reconstruction with the TMG flap is a valid option for patients with low Body Mass Index (BMI), while Deep Inferior Epigastric Perforator flaps should be considered for patients with a higher BMI. Autologous free fat transfer proves to be a safe and efficient treatment option in mild cases of Poland syndrome for male and female patients, in combination with or without implant based reconstructive surgery. Multicentre studies should be conducted to achieve higher case numbers of this rare disease and support clinical decisions with more data.

## 1. Introduction

Poland syndrome or Poland anomaly describes a unilateral partial or full aplasia of the pectoralis muscles, deformity, or absence of the costal rip cartilage II-V, hypoplasia or aplasia of the breast and athelia, combined with unilateral brachy syndactyly. Since this relatively rare diverse syndrome consists of multiple deformities in different grades, it is known today by the name of one of its first describers, the British surgeon Alfred Poland, although this eponym is not undisputed [1,2]. Commonly described with an array of other congenital chest wall deformities, its incidence ranges between 1:7.000–1:100.000, depending on the severity. Ribeiro et al. proposed a classification based on clinical and radiological findings to evaluate the grade of the deformity and its phenotype (Table 1) [3]. Poland syndrome is further predominately found in men and affects the right side of the body [4]. While the etiology remains unclear even today and familial inheritance is rare, interruption of the embryonic blood supply, as well as sporadic mutation and consequent bud line cell death, have been postulated as possible culprits [5,6]. Furthermore, the literature suggests drug abuse during pregnancy as another potential cause for the deformity [7,8]. Due to the different grades of severity and the complex combination of individual deformities, providing the adequate care for patients presenting with Poland syndrome is extremely challenging for surgeons. Whereas pediatric surgeons are often the first ones confronted with severe cases in young patients, the true degree of the deformity often presents in adolescent patients after puberty, when the hormonal changes in the male and female chest become apparent. This unfortunately aligns with the age where self-consciousness, social norms and body image play an important role for individuals, increasing the suffering of those affected by Poland syndrome [9].

One of the first described treatment options, the pedicled latissimus dorsi flap, is frequently used for reconstruction of the chest wall and breast deformity [10,11,12]. Several other surgical techniques are available for autologous breast reconstruction, using microsurgical techniques and free flaps such as the deep inferior epigastric artery perforator (DIEP), anterior lateral thigh (ALT), transverse myocutaneous gracilis (TMG) or transverse rectus abdominis myocutaneous (TRAM) flap [13,14,15]. Other available options for reconstruction are autologous free fat transfer and fat grafting or the reconstruction with synthetic materials such as individualized breast implants [16,17,18]. Unfortunately, each procedure has its risks and limitations which have to be well balanced, and a very individualized treatment concept is key in most cases. Since Poland syndrome presents itself in a variety of degrees, each technique has its benefits and drawbacks. Treating surgeons need to offer a wide array of treatment options and frequently a combination of different surgical techniques is necessary to achieve the desired results [19]. The highly specific treatment for patients due to the very unique baseline situation of each breast deformity represents an intricate problem for surgeons and makes it very hard to offer reproducible and reliable reconstructive results. This highly individual surgical treatment approach could be decrypted when taking patients gender and body mass index (BMI) into account. We propose that the inclusion of patient’s sex and BMI into the surgical plan can provide assistance and alleviate the decision-making process in each individual case.

The goal of this manuscript is to provide an overview of available treatment options for surgical breast reconstruction in the adolescent and adult patient population, in order to achieve reproducible, reliable and symmetric results. To our knowledge a distinct treatment algorithm focusing on not only clinical severity, but also sex and BMI to simplify available treatment decision pathways has not been proposed so far. The goal of this narrative review and retrospective case study is to help other care providers to find the right concept of reconstruction by offering an up-to-date treatment algorithm based on our institutional experience and the results and outcome data of our patient collective.

## 2. Materials and Methods

A retrospective chart review of all patients receiving surgical reconstruction for Poland syndrome related breast deformities was conducted at our institution between December 2011 and May 2020. Demographic data including age, sex, BMI, degree of Poland syndrome, surgical intervention, history of smoking, and follow-up time were collected and analyzed. Only patients older than 15 years of age, who received treatment at our department and had confirmed clinical signs of Poland syndrome Grade I–III, were included in the study. Patient with other unilateral breast asymmetries without any chest alterations or other signs of Poland syndrome were not included in our review. We then grouped the patients according to the received intervention to provide structural data for the reviewed surgical techniques. The data and our institutional experience were used to create a treatment algorithm to aid in the selection of treatment. The study was conducted according to the guidelines of the Declaration of Helsinki and approved by the Institutional Review Board.

## 3. Results

After performing a review of our institutional data, 22 Patients (72.7% female, 27.3% male) meeting the inclusion criteria were identified (Table 2). The average age at the time of the first surgery was 28.1 years (SD 10.2) and the average time of follow-up was 28 months (SD 34.4). One (4.5%) patient treated was diagnosed with a mild (Grade I), 10 (45.5%) patients with a severe (Grade II) and 11 (50%) with a very severe (Grade III) form of Poland syndrome. The mean BMI (kg/m^2^) at the time of surgery was 24.0 (SD 3.5) and seven patients were active smokers according to their medical records.

Reconstruction with a microsurgical free flap was performed in nine cases (TMG, DIEP), whereas two patients received a local flap (Latissimus dorsi flap). Six patients were treated with an implant-based breast reconstruction of which four individuals also received a symmetrization procedure (e.g., using a breast implant, mastopexy or reduction mammaplasty) for the non-affected side. Autologous free fat transfer (lipofilling) was performed in two patients as monotherapy, while in 15 cases patients received a combination of another surgical treatment, including free flaps, implants, etc. together with lipofilling (Table 3).

Patients treated with a combination of the named surgical techniques together with lipofilling received 1.7 fat transfers on average (Range 1–4) and the mean volume of fat injected overall was 205 mL (Range 30–720 mL). The most causes for implant removal were capsular contracture of at least one breast (6/9), implant rupture (1/9) and dissatisfaction with the shape of the breast (2/9).

The overall complication rate including major and minor complication was 45.5%. Half (5/10) of the complications observed were minor complications such as implant rippling, partial fat necrosis or a hypertrophic scar needing a revision. In five cases major complications such as hematoma of the breast (1/5) or the donor site (1/5), flap necrosis (2/5) and postoperative cellulitis and wound abscess (1/5) occurred (Table 4).

## 4. Discussion

### 4.1. Latissimus Dorsi Flap

**Figure 1:**
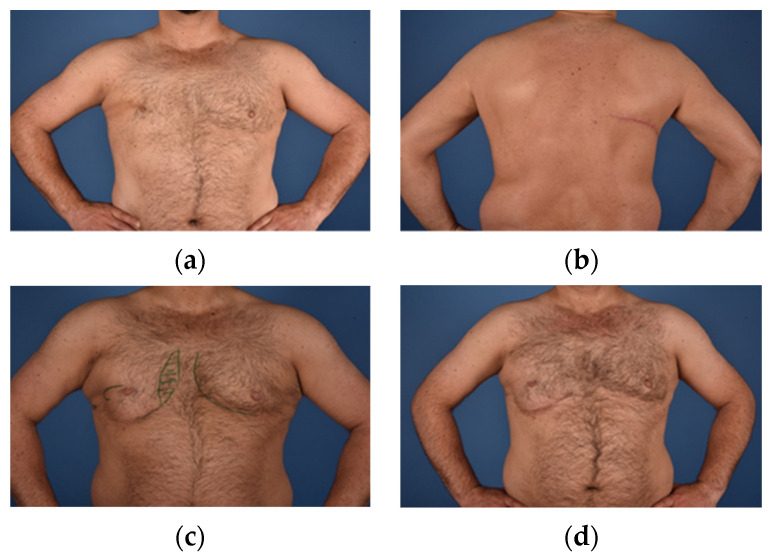
(**a**) = Male patient (40) pre-operatively with Grade II deformity; (**b**) = LDF donor site; (**c**) = Recipient site after LDF reconstruction (green lines mark fat grafting recipient sites); (**d**) = Recipient site after flap reconstruction and 2× autologous free fat transfer.

The Latissimus dorsi (LD) flap was one of the first attempts for autologous tissue reconstruction in patients with Poland syndrome [11,12]. Its proximity to the chest wall and breast, enable a pedicled ipsilateral flap reconstruction, which represents an advantage compared to more complex techniques, such as microsurgical free flap reconstructions and their potential risk for anastomosis related complications. The size and muscular tissue of the LD flap are usually sufficient to replace the absent major and minor pectoral muscles and offers an adequate compensation of the deformity [20]. While an adequate reconstruction and chest symmetry is often feasible for the male patient or even in slim/low BMI adolescent females, the possible muscular atrophy and consequently limited tissue volume recruitable for reconstruction, represents a drawback of this technique. Alterations and refinements of the LD flap were developed to reduce its volume limitations, including simultaneous lipofilling or a combination with silicone implants (Figure 1) [21,22].

To make up for the functional limitations of the fully or partially missing pectoral muscles, the transposed and still innervated LD muscle can even be trained to improve strength and function of the upper extremities [23,24]. While scarring and the potential functional deficit at the donor site are important factors to be considered when choosing the LD flap, new technologies, such as minimal invasive flap harvesting using robotic-assisted surgery, may offer improvements and new possibilities to improve this well-established technique further [25].

In our experience the LD flap represents a valuable reconstructive option for autologous breast reconstruction in male patients with a low-normal BMI suffering from Poland syndrome. Unfortunately, in low BMI females, the LD flap frequently does not offer enough soft tissue for symmetric breast reconstruction. Further, the relatively high donor site morbidity, together with the often times large donor site scar, does not justify the relatively small flap and later still present breast asymmetry. In our hands, even the named refinements such as lipofilling are sometimes not able to fully offer a suitable symmetric and satisfying result and cannot be offered in patients with a low BMI due to lack of tissue availability. 

Due to the potential need for silicone implant exchanges every decade and the risk of later capsular fibrosis the use of individualized breast implants in combination with the LD flap can hardly be recommended to male patients and does rarely represent the best option for breast reconstruction in this patient collective. Consequently, the innervated pedicled ipsilateral LD flap can only be recommended for breast reconstruction in low to normal BMI Poland syndrome patients and is able to achieve more favorable results in the male patient collective.

### 4.2. Transverse Myocutaneous Gracilis Flap

Since its first description by Yousif et al., the transverse myocutaneous gracilis (TMG) flap has become a commonly used free flap for breast reconstruction due to its versatility, constant and reliable anatomy and relatively simple flap harvest [26]. Although the DIEP flap remains to be the gold standard in autologous breast reconstruction for most plastic surgeons, the availability and reliability of the TMG flap in patients with a low BMI who do not offer enough tissue for an abdominal based reconstruction make the TMG flap a sound second choice for breast reconstruction in such patients [27]. Donor site morbidity of the thigh has been postulated as a drawback but has been shown to be comparable to abdominal based and other free flap options available [28,29].

**Figure 2:**
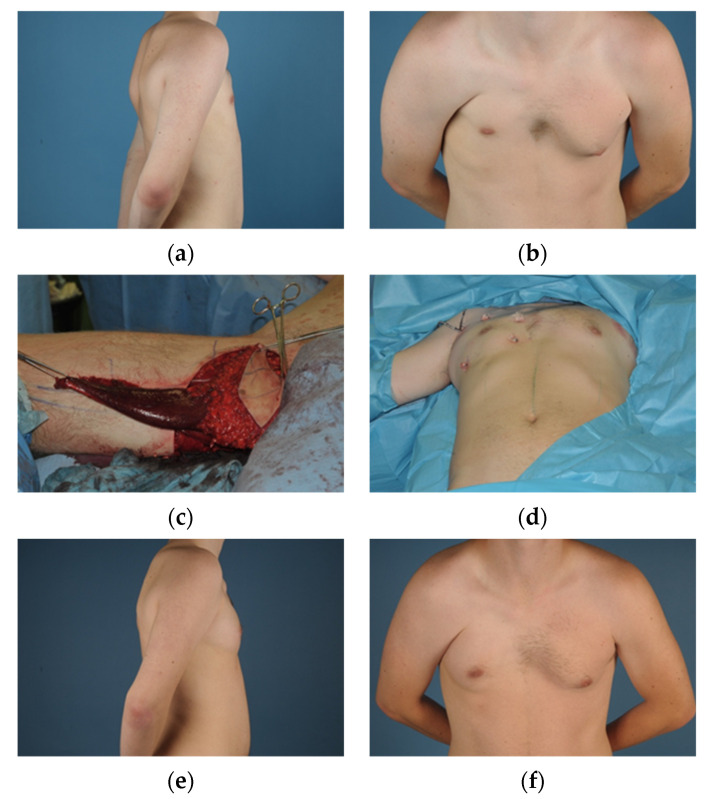
(**a**) = Male patient (21) pre-operatively with Grade III deformity, side view; (**b**) = front view; (**c**) = The ipsilateral TMG is prepared and the muscle resected at the distal insertion; (**d**) = After anastomosis to the thoracodorsal vessels and nerve, the borders of the flap are fixated through the skin; (**e**) = 8 months postoperative, side view; (**f**) = 8 months postoperative front view.

Huemer et al., reviewed 14 TMG flaps in 11 patients suffering from Poland syndrome and were able to show not only a low donor site morbidity and complication rate, but also a high patient satisfaction for their patients [13]. Another case series from *Wechselberger* et al. was capable to show similar results in three male patients who received chest wall reconstruction with the TMG flap to improve the form of the torso and correct the deformity of the axillary fold which can frequently not be addressed adequately using other techniques [30]. The TMG flap shows its strength in thin patients where volume for reconstruction is needed but scarce and it can function as a stand-alone treatment in male and female patients as well as in combination with a silicone implant or lipofilling [31]. Our institutional experience is that the TMG flap represents a very reliable technique for breast reconstruction in breast cancer patients and patients suffering from trauma or various other deformities likewise. The constant anatomy of the flap, comparably low donor site mobility and well concealed scar compared to other myocutaneous flaps, position the TMG as a valid soft tissue resource in both, male and female patients. While the relatively small size of the flap compared with the DIEP and the risk of volume loss due to muscle atrophy represent a drawback, our experience has shown that especially the combination with lipofilling can compensate these potential shortcomings (Figure 2).

Especially in male Poland syndrome patients with severe (Grade II) and very severe (Grade III) deformities, the TMG flap can be utilized as an innervated muscle flap when coapted to the thoracodorsal nerve in order to use it as a functional replacement for the missing pectoralis muscles (Video S1). Our study also included cases of female Poland syndrome patients who received breast reconstructions with TMG flaps. Since the TMG flap is usually considered a smaller sized flap in breast reconstruction with an average weight of 320 g, the average BMI for optimal treatment should be <25 kg/m^2^, although higher BMI is not a contraindication to utilization of the TMG flap [27,32]. However, in patients with a higher body weight, the DIEP flap which offers a mean average weight of 550 g is frequently a better choice for reconstruction since it offers more volume and many patients potentially benefit from the donor site in the form of an abdominoplasty [33].

**Figure 3:**
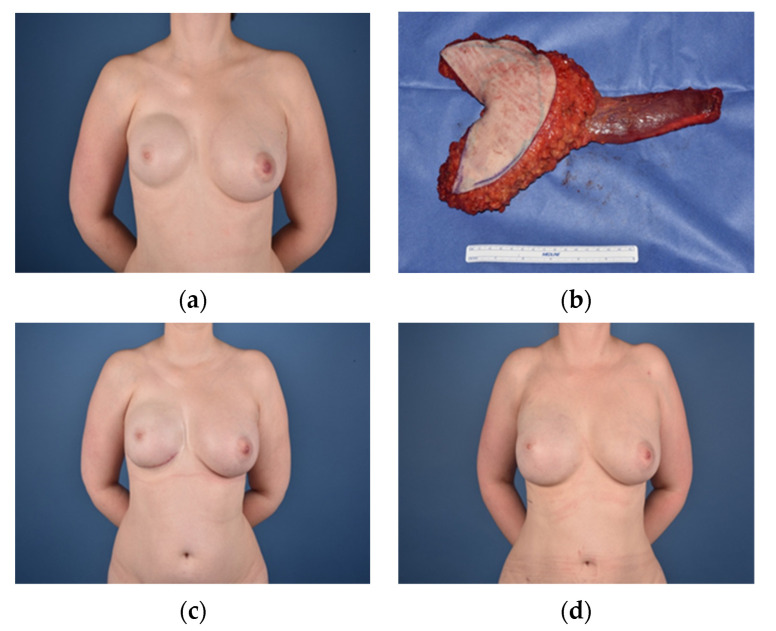
(**a**) = Female patient (27) pre-operatively with Grade II deformity and previous implant-based reconstruction; (**b**) = TMG flap before anastomosis; (**c**) = Recipient site 6 weeks after flap reconstruction; (**d**) = Recipient site 29 months after flap reconstruction and 4× autologous free fat transfer (720 mL total).

A unique advantage of the TMG flap in Poland syndrome reconstruction is the possibility to position the skin island in the lower breast pole where most of the volume is missing and reattach the gracilis muscle tendon on the humerus in order to reconstruct the normal pectoralis muscle insertion. Hereby, the TMG flap can be used to replace like with like, by reconstructing the insertion of the muscle on the humerus. Therefore, the deep indentation of the axillary fold, which is often times extremely hard to reconstruct and very stigmatising for patients, can be relined and rebuilt. The majority of our patients additionally received secondary lipofilling after their free flap reconstruction, to improve the missing volume of the anterior axillary fold to further undergird this area. The size of the skin island of the TMG flap usually provides enough surface area to allow for primary reconstruction, even in total aplasia of the breast. It is also a valuable option for secondary reconstruction after initial reconstruction with implants or prior expander treatment (Figure 3).

### 4.3. Deep Inferior Epigastric Perforator Flap

Although the deep inferior epigastric perforator (DIEP) flap strongly remains the most commonly used free flap for breast reconstruction, it is rarely found in the literature as a first line treatment for Poland deformity. This is surprising, considering its popularity and the plethora of scientific studies surrounding it. The benefits of the DIEP flap are its size, which helps especially in cases where the contralateral breast is fully developed and voluminous, as well as the often-appreciated abdominoplasty that comes with the donor site closure. Compared to the visible donor site scar of the LD flap, the abdominal scar is a favorable trade for patients. Since there is no muscle atrophy, the amount of tissue loss over time is also significantly lower, providing more steady and reliable results [34]. While the DIEP flap is best used in cases with a significant volume deficiency or asymmetry of the contralateral breast, the few reports that can be found in the literature show promising results and patient satisfaction [15,35]. Lack of abdominal tissue in thin patients poses a limiting factor for the DIEP flap, making it necessary for surgeons to offer alternative free flaps as a backup plan. For primary reconstruction and cases where implant based primary reconstruction failed, the deep inferior epigastric perforator flap can also provide a valuable option to the surgeon [36].

**Figure 4:**
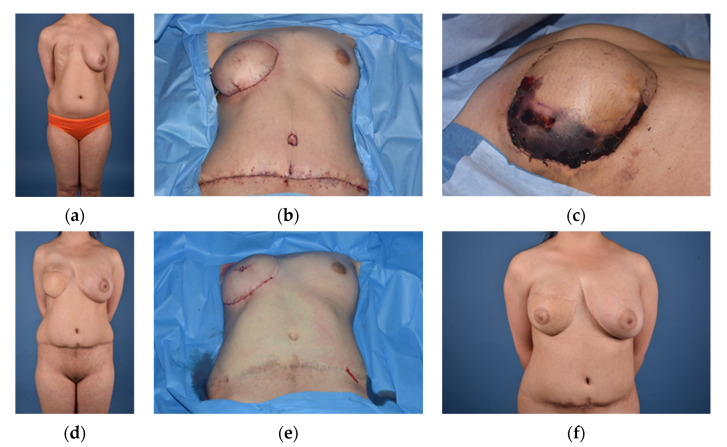
(**a**) = Female patient (24) with severe deformity and absence of breast and areola preoperatively; (**b**) = Postoperative image after DIEP flap reconstruction; (**c**) = Partial lateral necrosis of the flap; (**d**) = 3 months after flap reconstruction and necrectomy; (**e**) = Form correction of the breast, nipple reconstruction (arrow flap) and lipofilling (220 mL) after 13 months; (**f**) = Final result 21 months after DIEP flap, (areola tattoo after 15 months).

In our study we report three female patients who received unilateral reconstruction with a DIEP flap (Table 3). With an average BMI of 29.2 kg/m^2^, it was predominately used for overweight patients in our collective. Nevertheless, two of our patients also received additional lipofilling to improve the aesthetic outcome and symmetry, raising the point that volume alone is often not enough, if not in the right place (Figure 4). In patients with very large breasts, even the DIEP sometimes requires combination surgery of the contralateral side to improve the aesthetic outcome.

Although the DIEP flap does not qualify as a gold standard for the treatment of Poland breast deformity it represents an essential and unique option to reconstruct a large breast in patients with a higher BMI, which offer enough tissue resource in the lower abdomen. This is especially the case in female patients which benefit more from an abdominoplasty and fasciocutaneous breast flap breast reconstruction, compared to male patients which may benefit from a functional myofasciocutaneous flap breast reconstruction such as the TMG flap.

Breast reconstruction incorporates soft fatty- and gland-tissue in females, which can be offered using a DIEP flap. The male chest, from an aesthetic and functional point of view, benefits more when a myocutaneous flap reconstruction such as an LD or TMG flap is used to replace the missing pectoralis major muscle.

### 4.4. Implant Based Reconstruction

Compensating the missing volume of the chest wall and/or breast with an implant is one of the most frequently used techniques for treating patients with Poland syndrome. Different textures have been used over the last decade to improve the present structural deficit. While the results of customized implants are often aesthetically sound, the long-term effects of the foreign bodies and their inability to adapt to changes of the patient’s body proved to be a disadvantage [17,18,37,38,39]. Mispositioning and seroma are the most commonly occurring complications, and while early-stage seroma rates of up to 30% are found in the literature, there is little reported data on late onset chronic seroma [40,41]. In men, a customized silicone or other polymeric implant is often regarded a popular and sufficient treatment, resulting in a camouflaging of the chest wall defect and a negligible scar at the incision site [41,42].

**Figure 5:**
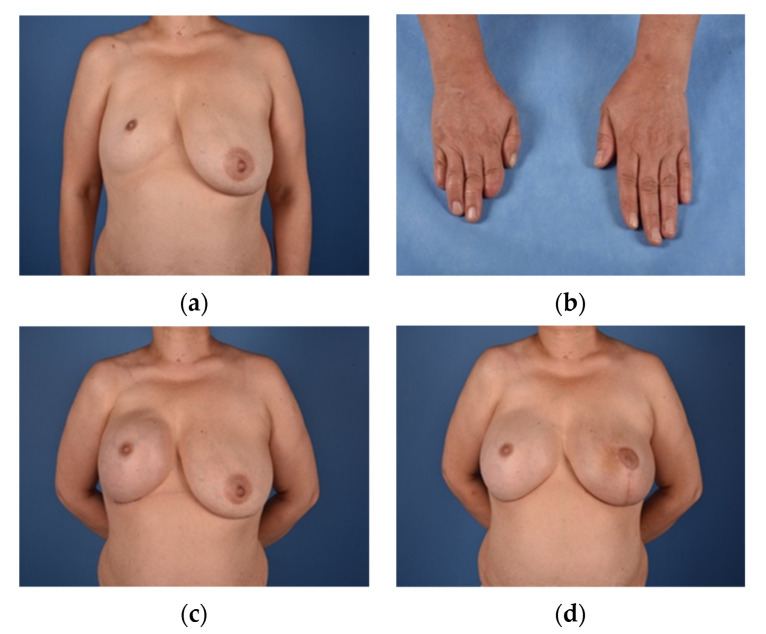
(**a**) = Female Patient (48) with Grade III deformity; (**b**) = Ipsilateral brachydactylia; (**c**) = Expander 5 months after implantation; (**d**) = Final result (8 months) after Implant reconstruction (485 cc), breast reduction mammoplasty of the contralateral breast and singular lipofilling (200 mL).

A risk and benefit stratification comparing free flaps and the risk for complications and other drawbacks, such as potential atrophy of the donor muscle, donor site complications and longer surgical times needs to be compared to customized implants in each individual case [43,44]. In young patients, customized implants are a suitable option for treating the chest wall defect, while changes of the chest and breast during adolescence can impact implant position and potentially alter the result and symmetry [44,45]. Primary implantation of a tissue expander is often needed to ensure enough skin coverage of the implant in female patients (Figure 5). In severe (Grade II) and very severe (Grade III) Poland deformities of female patients, the underlying musculoskeletal deformity is often not sufficiently treated with an implant alone. Combinations of pedicled myocutaneous flaps such as the LD in combination with an implant, represent a frequently used option for reconstruction in female patients with a low to normal BMI [46,47]. Insertion of silicone implants is seen as a safe procedure to compensate breast asymmetry in cases with none to mild chest deformity, but patients have to be informed about the possible risk and long-term implications, such as capsular contracture and the lifelong need for potential implant exchange [43].

In our experience the use of implants to reconstruct Poland syndrome related deformities needs to be very carefully considered and all alternative autologous reconstructive techniques need to be discussed with patients in order to offer an individually tailored surgical treatment for each patient. Additional lipofilling on the affected side or lipofilling/breast reduction mammoplasty on the contralateral side are mostly needed to achieve individually pleasing aesthetic results. Again, a patient’s gender and BMI can assist surgeons choosing the right option and either opting for or against an implant reconstruction. While patients with a higher BMI can potentially camouflage implant edges due to increased soft tissue cover, patients with a low BMI can have a foreign body feeling or suffer from rippling and palpable or visible implant edges together with a potentially unsatisfying symmetry.

### 4.5. Autologous Free Fat Transfer

**Figure 6:**
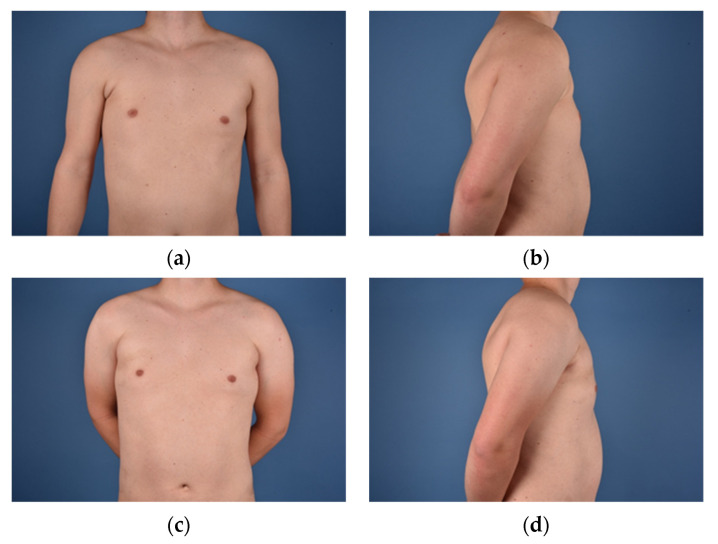
(**a**) = Male patient (25) with Grade I deformity; (**b**) = Preoperative side view; (**c**) = 17 months after 2× lipofilling (220 mL total); (**d**) = Postoperative side view.

Autologous free fat transfer (lipofilling) is commonly used today as a stand-alone treatment for structural tissue deficiencies or in combination with other procedures such as implants or pedicled and free flap reconstructions. Especially in mild cases of uni- or bilateral hypomastia, lipofilling has demonstrated its potential and benefits (Figure 6) [48,49,50]. While the procedure is low in risk and often appreciated for the aesthetic benefits of a liposuction at the donor region, the usual need for several treatments to achieve satisfying results is a drawback and patients need to be made aware of the necessary patience in order to achieve a symmetric result. A series of eight patients by Pinsolle et al. reported 1–5 lipofillings necessary in combination with other procedures such as implants or flaps, in order to achieve sufficient volume and symmetry [16]. While lipofilling alone might be sufficient only in mild cases, it still has its role in the treatment of the more severely afflicted, by combining it with other procedures (Figure 1, Figure 3, Figure 4 and Figure 5) [51,52]. The disadvantages of size and atrophy in muscular flaps for example can be safely compensated by additional autologous fat transfer [31,53]; 77.3% of our reported cases received at least one treatment of lipofilling with an average volume of 205 mL.

For implant-based reconstructions, it can be used to increase the size of the pectoralis muscle, if existing, by direct injection or to improve the thickness of the skin covering the implant [54]. Again, gender and patient’s BMI can guide surgeons towards the use and indication of lipofilling to treat Poland syndrome deformity. In our experience, especially female patients with a normal to high BMI benefit from lipofilling, however even male patients with a similar BMI pattern can benefit using lipofilling techniques.

## 5. Conclusions

**Figure 7:**
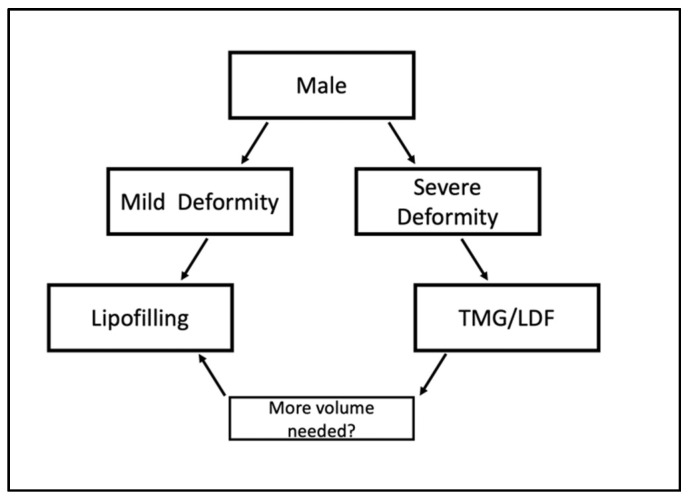
Algorithm for treatment of male patients.

In regard to our institutional experience and the data presented, we want to provide a guideline for surgeons to assist in considering possible treatment options for patients with Poland syndrome. Since male and female patients differ in the requirements to their breasts, we offer an algorithm for each sex. In our experience male patients with mild deformities benefit from treatment with lipofilling, to address the missing breast volume and deformity of the axillary fold (Figure 7).

In more pronounced cases, reconstruction with a myocutaneous flap should be considered to create enough volume and then improved by secondary lipofilling if needed.

**Figure 8:**
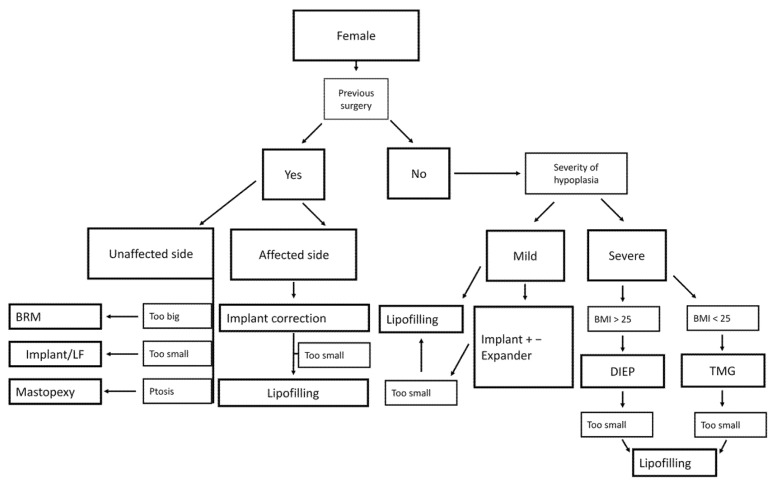
Algorithm for treatment of female patients.

As described previously, sufficient reconstruction of the female breast has proven to be more challenging due to the complex structure and higher aesthetic expectations of the patients. Since age, pregnancy and weight changes directly affect the breast tissue and size, providing correction procedures of the affected and non-affected breast is usually needed after a certain time. In our institutional experience, patients who received previous implant-based reconstruction were highly satisfied with implant changes and additional correction procedures such as lipofilling, mastopexy, or breast reduction surgery. For mild cases, implants with or without additional lipofilling can produce satisfying results, but it should be noted to thoroughly inform the patient about the potential necessity of the above-mentioned correction procedure in the long run (Figure 8).

Regarding primary reconstruction in more severe cases, a BMI based approach proved helpful in our experience. Since a higher BMI is frequently accompanied with an increased breast size, these patients benefit from the larger volume flaps such as the DIEP flap, while simultaneously benefitting from the abdominoplasty of the donor site. The TMG flap shows its advantages in the slim and athletic, female and male patient population and can be combined with lipofilling if needed to allow for a more refined and symmetrical result.

While our institutional experience with the proposed algorithms is good, we see a personalized treatment plan for each patient presenting with this complex deformity as the key to success. Since it is rare in its occurrence, scientific papers with numerous patients are rare and further studies, especially regarding patient outcome and satisfaction, are needed. We still hope that our manuscript can contribute another helpful guide to help other surgeons provide for their patients.

## Figures and Tables

**Table 1:** Poland Syndrome Classification (modified from Ribeiro et al. [3]).

Mild (Grade I)	Amastia; hypomastia or areolar asymmetry No musculoskeletal chest alterations or partial absence of pectoralis major muscle No superior limb alterations Other congenital alterations may be present
Severe (Grade II)	Hypomastia or amastia; areolar asymmetry Total absence of pectoralis major muscle; different alterations of the ipsilateral bones/muscles of the chest No superior limb alterations Other congenital alterations may be present
Very Severe (Grade III)	Amastia; areolar asymmetry Different musculoskeletal alterations Ipsilateral superior limb alterations Other congenital alterations may be present

**Table 2:** Patient overview.

Characteristic	Number				%
Cases included	22				100
Sex					
Male	6				27.3
Female	16				72.7
Age, years			male	female	
Mean		28.1	24.1	29.6	
SD		10.2	8.3	10.7	
Follow-Up, months					
Mean		28.0			
SD		34.4			
BMI, kg/m2			male	female	
Mean		24.0	24.5	23.7	
SD		3.5	2.1	4.0	
Smoker					
Yes	7				31.2
No	15				68.8
Severity					
Grade I	1				4.5
Grade II	10				45.5
Grade III	11				50.0

**Table 3:** Overview and clinical presentation of patients.

Patient	Sex	Age Years	Grade	BMI	Side	Previous Treatment	Surgical Treatment	LF No.	Volume mL
1	F	16	III	21.3	R	Expander	Implant (600 cc)	-	
2	F	37	III	19.8	R	LDF + Implant	Implant (400 cc)	2	245
3	F	14	II	22.3	R	Expander	Implant (400 cc)	1	60
4	F	38	II	26.0	R	LDF + Implant	Implant (280 cc)	1	50
5	F	48	III	27.4	R	Expander	Implant (485 cc) + BRM	1	200
6	F	18	II	21.4	L	None	Implant (255 cc) + BRM	-	
7	F	35	II	20.5	L	Implant	Implant (180/245 cc) + Mastopexy	-	
8	F	25	II	22.1	R	Implant	Implant (160 cc) + Mastopexy	1	110
9	F	42	II	23.3	L	Implant	Implant (175 cc) + Mastopexy	1	60
10	M	21	III	22.4	R	None	TMG	-	
11	M	22	III	23.8	R	None	TMG	2	130
12	M	20	III	22.6	L	None	TMG	3	90
13	F	18	III	20.1	R	None	TMG	1	100
14	F	27	II	27.0	R	Implant	TMG	4	720
15	F	43	II	22.3	R	Custom Implant	Double TMG	4	520
16	F	24	III	24.5	R	None	DIEP	1	220
17	F	32	III	28.3	R	None	DIEP	1	30
18	F	36	III	34.7	L	LDF + Implant	DIEP + BRM	1	400
19	F	20	III	21.3	L	Implant	LDF	-	
20	M	40	II	27.5	R	None	LDF	2	140
21	M	17	II	26.6	R	None	Lipofilling	2	330
22	M	25	I	23.8	R	None	Lipofilling	2	220

LF = Lipofilling; DIEP = Deep inferior epigastric perforator flap; TMG = Transverse myocutaneous gracilis flap; LDF = Latissimus dorsi flap; BRM = Breast reduction mammoplasty.

**Table 4:** Overview of complications.

		Implant *n* = 9	DIEP *n* = 3	TMG *n* = 6	Latissimus *n* = 2	Lipofilling *n* = 2
Minor complication *		1	1	2	1	-
Major complication		-	-	-	-	-
Hematoma breast		1	-	-	-	-
Hematoma donor site		-	-	1	-	-
Flap necrosis		-	2	-	-	-
Cellulitis/Abscess		-	-	1	-	-

* Scar needing corrective surgery, Implant rippling, Partial fat necrosis.

## Supplementary Materials

The following are available online at https://www.mdpi.com/article/10.3390/jcm10194515/s1, Video S1: Functional free TMG Flap in Poland Syndrome Breast Reconstruction in a Male Patient.
